# Refinement of Hyams Grading Criteria for Olfactory Neuroblastoma in an Endoscopic Sinus Surgery Predominant Cohort with Extended Follow-up: Worth the Effort?

**DOI:** 10.1007/s12105-025-01804-z

**Published:** 2025-07-29

**Authors:** Stacey M. Gargano, Vincent Cracolici, Carl H. Snyderman, Eric W. Wang, Garret Choby, Aron Z. Pollack, Paul A. Gardner, Diana Bell, Raja R. Seethala

**Affiliations:** 1 Department of Pathology and Genomic Medicine, Thomas Jefferson University, 132 S. 10th St, Suite 285Q, Philadelphia, PA 19107 USA; 2 Department of Pathology and Laboratory Medicine, Cleveland Clinic, Cleveland, OH USA; 3 Department of Otolaryngology, University of Pittsburgh, Pittsburgh, PA USA; 4 Department of Otolaryngology, Northwell University, New York, NY USA; 5 Department of Neurosurgery, University of Pittsburgh, Pittsburgh, PA USA; 6 Department of Pathology and Laboratory Medicine, University of Pittsburgh, Pittsburgh, PA USA

**Keywords:** Case-control studies, Disease progression, Esthesioneuroblastoma, Olfactory* / mortality, Esthesioneuroblastoma, Olfactory* / pathology, Neoplasm grading, Neoplasm staging, Nose neoplasms* / pathology, Retrospective studies, Survival analysis

## Abstract

**Background:**

Hyams grading is considered prognostic in olfactory neuroblastoma (ONB), but grading criteria are subjective and predate modern classification and surgical approach. We evaluate the application of granular grading criteria to an endoscopic surgery predominant cohort with extended follow-up.

**Methods:**

78 ONB patients were identified (1994–2019) with original diagnoses dating to 1979. Original Hyams grade, Modified Hyams grade incorporating more detailed criteria and other histologic features (i.e. divergent differentiation, clear cell/oncocytic change, and spindling) were assessed if feasible, evaluated for distribution by grade and correlated with outcomes.

**Results:**

Original Hyams grade (*n* = 43) distribution was: I: 4 (9%), II: 27 (63%), III: 11 (26%), and IV: 1 (2%). Modified Hyams grade (*n* = 59) distribution was: II: 29 (49%), III: 26 (44%), and IV: 4 (7%) with no grade I cases. Cases were more frequently upgraded with Modified Hyams grade. Mitotic rate (adjusted *p* < 0.001), pleomorphism (*p* < 0.001) and absence of rosettes (*p* = 0.002) were the only features that varied significantly between low grade (I-II) and high grade (III-IV) ONB. Neither Original nor Modified Hyams grade correlated significantly with any outcome endpoints by univariable Cox regression analysis (Median follow-up on surviving patients: 121.8 months). Splitting Modified Hyams grade into low and high trended towards a significant association with disease free survival on Kaplan Meier analysis (DFS, log rank *p* = 0.088). Severe nuclear pleomorphism correlated with adverse disease specific survival (Hazard ratio (HR): 8.092, *p* = 0.024) and DFS (HR:3.81, *p* = 0.033). On multivariable Cox regression, only procedure type (combined and transcranial approaches) remained significant prognosticators [HR:8.616, *p* = 0.004, and HR: 17.559, *p* = 0.017, respectively] with Kadish-Morita Stage D being nearly significant [HR:14.35, *p* = 0.054].

**Conclusion:**

Modified Hyams grade offers a slight improvement over Original Hyams grade but is still not particularly impactful as compared to disease extent/stage and procedure type. Hyams grade I and IV are rare and likely unnecessary, justifying resolution of ONB grading to simply high and low grade. Furthermore, it appears that only a subset of parameters for Hyams grade is relevant in terms of classification and outcome.

## Introduction

Olfactory neuroblastoma (ONB) was first characterized by Berger, Luc and Richards in 1924 [1] and is a neuroectodermal tumor of the sinonasal tract, arising from the olfactory neuroepithelium of the nasal vault and accounting for approximately 3% of all sinonasal tract neoplasms [2]. There is a slight male predominance and wide age range (2–90 years) with the incidence peaking in the 5th -6th decades of life [2, 3]. The earliest clinical manifestations of ONB include nasal obstruction and/or epistaxis, with other symptoms depending on the extent of disease [2]. ONB may also rarely show an association with paraneoplastic syndromes [4]. The classic radiologic appearance is that of a dumbbell-shaped mass centered at the cribriform plate, with its lower portion in the upper nasal cavity and its upper portion in the intracranial fossa [5]. Histologically, ONB is a prototypically small round blue cell tumor characterized by a lobular growth pattern, prominent intralobular fibrovascular stroma, and variable neurofibrillary matrix. The ONB immunoprofile typically consists of strong neuroendocrine marker expression (i.e. synaptophysin, neuron specific enolase, chromogranin, and INSM-1), a sustentacular pattern of S100 staining, and negativity for cytokeratins [2].

With the advent of combined multimodal therapy (surgery + chemoradiation), outcomes for ONB have improved drastically over the past century with 5- and 10-year disease specific survival as high as 94% and 89%, respectively [6, 7]. However, key determinants of prognosis remain the extent of disease and morphologic features. Several key staging systems putting a framework to extent of disease have been applied to ONB for at least one half of a century with varying degrees of success [7–12].

Along similar lines, ONB had been stratified into a four-tiered grading system (I-IV) by Hyams [13] in 1982 based on a combination of growth pattern, matrix characteristics, rosette types, nuclear pleomorphism, mitoses, necrosis, and calcification. While several series have demonstrated the prognostic value of Hyams grade, either alone or combined with stage [3, 7], this finding is not consistent [11, 14]. In more recent studies [3, 6, 7], the functionality of Hyams grade has been improved by reducing it to two tiers: Hyams low (I-II) and Hyams high (III-IV) grade.

Nonetheless, several limitations to Hyams grade remain:

1. This system predates immunohistochemistry, and many if not most tumors that were historically grade IV likely represent other entities altogether.
2. The system is purely qualitative and inherently imprecise.
3. Parameters in Hyams grade have not been evaluated for relative contribution to overall grade and prognosis.

To address these issues, we have developed a semiquantitative modified version of the Hyams grading system for ONB and evaluated each parameter’s contribution to defining grade as well as prognosis in the context of an endoscopic endonasal and skull base surgery predominant cohort with extensive follow-up.

## Materials and Methods

This study was approved by our Institutional Review Board (IRB) through the University of Pittsburgh Human Research Protection Office (STUDY20040320).

### Case Selection

All in-house cases of ONB for which diagnostic material was reviewed at our institution (1994–2019) were included. If initially seen as a recurrence, initial diagnostic slides and/or reports were searched via paper archive dating back as early as 1979. Subsets of this retrospective cohort were included in prior studies [6, 7, 15]. Clinical and pathologic parameters were updated and clarified as needed.

### Morphologic Analysis

Two to four pathologists (S.G and/or V.C, D.B, and R.R.S) confirmed the diagnosis of ONB using the criteria:

1. Nasoethmoid location.
2. Characteristic morphology (round blue cell appearance, lobular growth pattern, prominent intralobular fibrovascular stroma, neurofibrillary matrix).
3. Exclusion of other round blue cell entities by ancillary studies and/or establishing the typical ONB immunophenotype (synaptophysin, neuron specific enolase, and/or chromogranin positivity, S100 or SOX-10 sustentacular staining, and negativity or at most focal staining for cytokeratin). Keratin reactivity was accepted in ONB with divergent epithelial differentiation (i.e. olfactory carcinoma).

The Original Hyams [13] grade was extracted from the original report. A Modified Hyams grade was derived from the Original Hyams grade parameters by two pathologists (S.G. and R.R.S.) and summarized in Table 1. Each parameter (architecture, neurofibrillary matrix, rosettes, mitotic activity, nuclear pleomorphism, necrosis and calcification) was defined more granularly and quantitatively as applicable. Grade was assigned based on majority distribution of the parameters. Additional features (perineural, lymphatic/vascular, and microscopic bone invasion, spindling, clear cell/oncocytic change, epithelial interaction, and ganglioneuromatous stroma) were documented as well. ‘Epithelial interaction’ was a parameter created to assess potential morphologic transitional forms towards divergent epithelial differentiation. It has been defined by a spectrum of epithelial components that includes gland entrapment without or with proliferative epithelial changes and/or mucosal surface involvement. Overt divergent epithelial differentiation by morphology and/or immunohistochemistry was recorded separately.

**Table 1:** Description of original and modified Hyams grade parameters

Original Hyams Grade					Modified Hyams Grade			
I	II	III	IV		I	II	III	IV
Lobular	Lobular	±Lobular	±Lobular	**ARCHITECTURE**	> 75% lobules (fit in 10x field)	> 25% small lobules (fit in 20-40x field)		> 5% frank infiltration
Prominent	Present	May be present	Absent	**MATRIX**	> 25% of surface area	< 25% of surface area		None
Absent to slight	Present	Prominent	Marked	**PLEOMORPHISM**	< 2:1 nuclear variation		2-<4: nuclear variation (> 50% of tumor)	≥ 4:1 nuclear variation (> 10% of tumor)
HR	HR	FW	FW	**ROSETTES**	Pinealocytomatous	HW	FW	None
Absent	Present	Prominent	Marked	**MITOSES (per 2 mm** ^**2**^ **)**	0	1–2	3–9	≥ 10
Absent	Absent	Present	Prominent	**NECROSIS**	None		Punctate	Confluent
Variable	Variable	Absent	Absent	**CALCIFICATION**	Present		Absent	

HW: Homer Wright, FW: FlexnerWintersteiner.

### Clinical Features

The surgical procedures were stratified as follows: endoscopic endonasal approach (EEA), open transfacial resection, transcranial approach, and combined approach (EEA and transcranial). Maximal extent of the tumor (whether confined to sinonasal tract or extending to another anatomic site) and sinonasal compartments involved were documented based on a combination of clinical, radiologic findings and pathologic specimen site designations. All cases were assigned a Kadish-Morita Stage defined as follows: A (tumor limited to nasal cavity), B (tumor involves nasal cavity and paranasal sinuses), C (tumor extends beyond paranasal sinuses) or D (regional or distant metastasis) [12].

### Statistical Analysis

Statistical analyses were performed using R programming language [R Core Team (2023), R: A language and environment for statistical computing. R Foundation for Statistical Computing, Vienna, Austria, https://www.R-project.org/, R version 4.3.1 (2023-06-16)]. Key packages utilized included: tidyverse [16], survival [17], ggsurvfit [18], coxphf [19], flextable [20], and officer [21].

Categorical histologic variables were compared for low grade vs. high grade by Fisher exact test (2 × 2) or the Fisher Freeman Halton exact test (r x c), with the latter being subject to a Monte Carlo Simulation (*n* = 200,000). Comparison of mitotic counts for low grade vs. high grade was done using a nonparametric (Mann-Whitney U) test. P values were adjusted using the Bonferroni method and values less than 0.05 were considered significant.

Survival analysis was performed for overall survival (OS), disease specific survival (DSS), and disease-free survival (DFS) using Kaplan-Meier estimates with log rank tests as well as univariable Cox regression with Firth’s penalized likelihood. For DFS, a multivariable model was constructed using the variables that were significant on univariable analysis, excluding confounding variables. The proportional hazards assumptions for the multivariable model were evaluated using Schoenfeld residuals. AIC and BIC criteria were used to select the final model.

## Results

### Demographic and Clinical Characteristics

78 patients were identified. Patient characteristics are displayed in Table 2 with the denominator for each variable restricted to those with available data. The mean and median ages at presentation were similar at 50 years (range: 13–89) and 50.5 years (interquartile range: 43.25–59.75) respectively. A male predilection (F: M = 0.66) was noted; and the majority (68/76, 89%) presented to our institution with primary disease. Most patients (48/64, 75%) were treated surgically by EEA alone and received adjuvant radiotherapy (61/75, 81%), with only 13% receiving neoadjuvant radiotherapy.

**Table 2:** Patient characteristics and clinicopathologic features

Parameter	Mean (Range)	Median (IQR)
**Age** (*n* = 78)	50 (13–89)	50.5 (43.25–59.75)
	***Subgroup n***	***Ratio or Proportion***
**Sex** (*n* = 78)		
Female	31	F: M = 0.66
Male	47	
**Status on Initial Presentation** (*n* = 76)		**%**
Distant metastasis	1	1
Primary	68	89
Recurrence	7	9
**Procedure** (*n* = 64)		**%**
EEA	48	75
Open	6	9
CA	8	12
TA	2	3
**Original Hyams Grade** (*n* = 43)		**%**
I	4	9
II	27	63
III	11	26
IV	1	2
**Modified Hyams Grade** (*n* = 59)^**@**^		**%**
II	29	49
III	26	44
IV	4	7
**Kadish-Morita Stage** (*n* = 69)		**%**
A	4	6
B	13	19
C	45	65
D	7	10
**Nodal Disease on Presentation** (*n* = 68)		**%**
No	62	91
Yes	6	9
**Number of Sinonasal Compartment Types** (*n* = 67)		**%**
1	8	12
2	23	34
3	26	39
4	10	15
**Maximal Extent** (*n* = 69)		**%**
Confined to sinonasal tract	21	30
Early skullbase	8	12
Orbit	3	4
Intradural	27	39
Cutaneous and soft tissue	2	3
Brain	8	12
**Neoadjuvant Radiotherapy** (*n* = 76)		**%**
No	66	87
Yes	10	13
**Adjuvant Radiotherapy** (*n* = 75)		**%**
No	14	19
Yes	61	81

^@^No case was Modified Hyams Grade I

EEA: endoscopic endonasal approach, CA: combined approach, TA: transcranial approach

The majority were either Kadish Stage B or C (13/69, 19%; and 45/69, 65% respectively). As such, tumors commonly (59/67, 88%) involved at least two sinonasal compartments. A significant proportion (27/69, 39%) had intradural extension. Extension into brain or cutaneous/soft tissue was noted in 12% (8/69) and 3% (2/69), respectively. About 9% (6/68) had nodal disease on presentation.

### Grade and Other Histologic Features

Original Hyams grade was assigned in only 63% (43/68) of primary ONB. Modified Hyams grade could be assigned in 87% (59/68). The majority under both original and modified Hyams grade were grades II or III (38/43, 88% for original grade, and 55/59, 93% for modified grade). Notably under Modified Hyams grade, there were no cases assigned grade I.

**Fig. 1:**
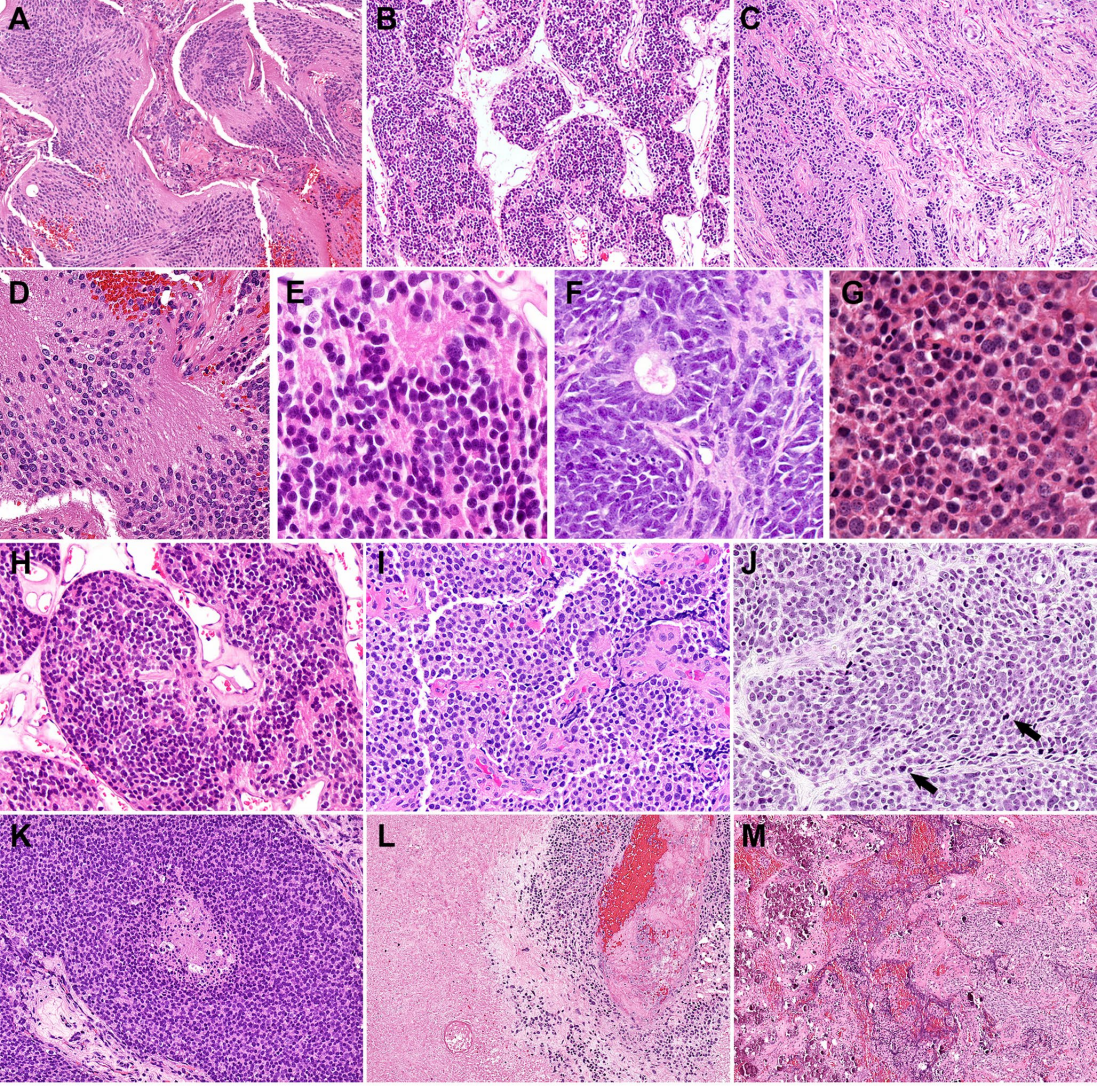
Morphologic features for Hyams grade parameters. **A**-**C**: Lobule and matrix characteristics. **A**- Large confluent fibrillary matrix rich lobules (10x). **B**- Smaller lobules with < 25% matrix (10x) C Infiltrative growth. **D**-**G**: Rosette types. **D**- Pinealocytomatous pseudorosettes (20x). **E**- Homer Wright pseudorosettes (40x). **F**- Flexner-Wintersteiner rosettes (40x). **G**- No rosettes or matrix (40x). **H**-**J**: Degrees of nuclear pleomorphism. **H**- Isomorphic with size variation < 2:1 (20x) I- Modest atypia with size variation ~ 2-<4:1 (20x). **J**- Severe atypia with nuclear size variation ≥ 4:1 (arrows– mitoses). **K**-**L**: Degrees of necrosis. **K**- Punctate (25x). **L**- Confluent (25x). **M**: Calcifications (10x)

Figure 1 shows select cases displaying the spectrum of histologic maturation that was used to help define the grading categories for each histologic feature. Original Hyams grade distribution (in the 43 cases where it had been assigned at the time of initial diagnosis) was: I: 4 (9%), II: 27 (63%), III: 11 (26%), and IV: 1 (2%). Modified Hyams grade distribution on 59 cases was: II: 29 (49%), III: 26 (44%), and IV: 4 (7%) with no grade I cases.

**Fig. 2:**
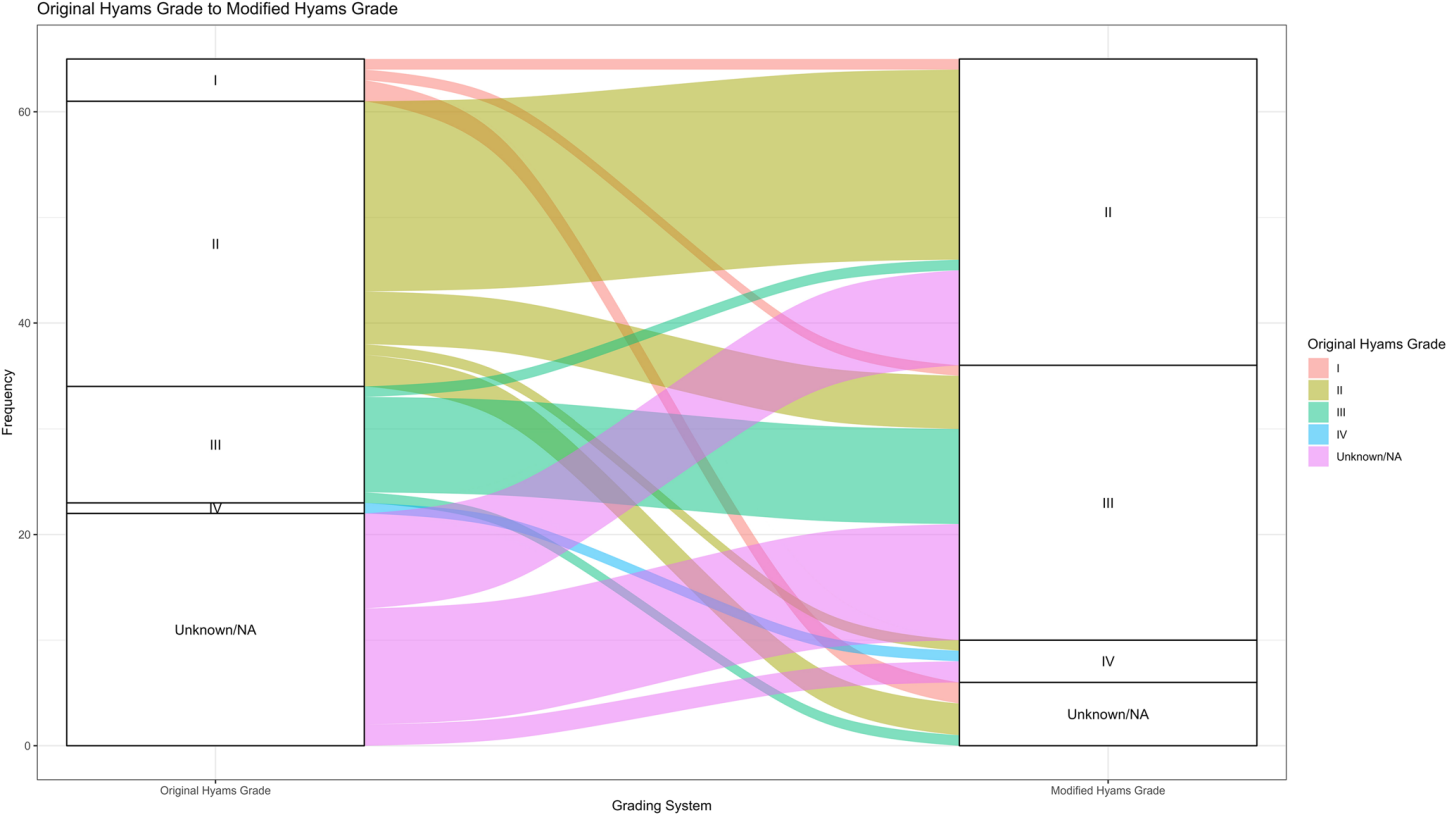
Alluvial chart demonstrating flow of cases from original Hyams grade to modified Hyams grade. Only one case was downgraded by the Modified Hyams grade (III-> II) while 8 were upgraded with two being two-step upgrades (I -> III, and II-> IV)

Figure 2 depicts the flow of cases for which either Original Hyams or Modified Hyams grade were available (*n* = 65). Twenty-two cases did not have an Original Hyams grade assigned, however these were assigned a modified Hyams grade on histologic review. Conversely, six cases for which there was an original Hyams grade could not be reviewed and assigned Modified Hyams grade. For the 37 cases for which both systems were applied, only one case (3%) was downgraded by the Modified Hyams grade (III-> II). Thus, 8/37 (22%) were upgraded with two (5%) being two-step upgrades (I -> III, and II-> IV).

Components of Hyams grade and other histologic parameters are stratified by grade grouping in Table 3. For grading parameters, only mitotic rate, pleomorphism and rosette type (specifically the presence or absence of rosettes) varied significantly between low- and high-grade groups.

**Table 3:** Histopathologic features

Parameter	*Low Grade (I-II)* (*n* = 29)	*High Grade (III-IV)* (*n* = 30)	*All* (*n* = 59)	*p*.adjusted
**Architecture** ^**#**^				1
> 25% small lobules	27/29 (93.1%)	28/30 (93.33%)	55/59 (93.22%)	
> 5% frank infiltration	2/29 (6.9%)	2/30 (6.67%)	4/59 (6.78%)	
**Mitoses per 2 mm** ^**2**^				**< 0.001**
Mean(Range)	1.52 (0–6)	8.33 (0–50)	4.98 (0–50)	
Median(IQR)	1 (0–2)	5 (3–8)	3 (1–5)	
**Necrosis**				0.819
None	24/29 (82.76%)	17/30 (56.67%)	41/59 (69.49%)	
Punctate	5/29 (17.24%)	12/30 (40%)	17/59 (28.81%)	
Confluent	0/29 (0%)	1/30 (3.33%)	1/59 (1.69%)	
**Nuclear Pleomorphism**				**< 0.001**
Mild	17/29 (58.62%)	3/30 (10%)	20/59 (33.9%)	
Moderate	12/29 (41.38%)	19/30 (63.33%)	31/59 (52.54%)	
Severe	0/29 (0%)	8/30 (26.67%)	8/59 (13.56%)	
**Nucleoli**				0.494
Indistinct	18/29 (62.07%)	10/30 (33.33%)	28/59 (47.46%)	
Prominent	11/29 (37.93%)	20/30 (66.67%)	31/59 (52.54%)	
**Vesicular Chromatin**				1
No	21/29 (72.41%)	19/30 (63.33%)	40/59 (67.8%)	
Yes	8/29 (27.59%)	11/30 (36.67%)	19/59 (32.2%)	
**Fibrillary Matrix** ^**#**^				0.312
≤ 25% of surface area	29/29 (100%)	24/30 (80%)	53/59 (89.83%)	
None	0/29 (0%)	6/30 (20%)	6/59 (10.17%)	
**Rosette Type** ^**#**^				**0.002**
Homer Wright	27/29 (93.1%)	13/30 (43.33%)	40/59 (67.8%)	
Flexner-Wintersteiner	0/29 (0%)	3/30 (10%)	3/59 (5.08%)	
None	2/29 (6.9%)	14/30 (46.67%)	16/59 (27.12%)	
**Calcifications**				1
Yes	9/29 (31.03%)	6/30 (20%)	15/59 (25.42%)	
No	20/29 (68.97%)	24/30 (80%)	44/59 (74.58%)	
**Spindle Cells**				0.13
No	27/29 (93.1%)	19/30 (63.33%)	46/59 (77.97%)	
Yes	2/29 (6.9%)	11/30 (36.67%)	13/59 (22.03%)	
**Clear Cells/Oncocytes**				1
No	13/29 (44.83%)	15/30 (50%)	28/59 (47.46%)	
Yes	16/29 (55.17%)	15/30 (50%)	31/59 (52.54%)	
**Epithelial Interaction / Differentiation**				1
None	20/29 (68.97%)	13/30 (43.33%)	33/59 (55.93%)	
Gland Entrapment	6/29 (20.69%)	10/30 (33.33%)	16/59 (27.12%)	
Gland Entrapment, Proliferative	2/29 (6.9%)	4/30 (13.33%)	6/59 (10.17%)	
Gland Entrapment, Surface	1/29 (3.45%)	0/30 (0%)	1/59 (1.69%)	
Divergent Epithelial Differentiation	0/29 (0%)	3/30 (10%)	3/59 (5.08%)	
**Ganglioneuromatous Stroma**				1
No	27/29 (93.1%)	28/30 (93.33%)	55/59 (93.22%)	
Yes	2/29 (6.9%)	2/30 (6.67%)	4/59 (6.78%)	
**Perineural Invasion**				1
No	21/29 (72.41%)	18/30 (60%)	39/59 (66.1%)	
Yes	8/29 (27.59%)	12/30 (40%)	20/59 (33.9%)	
**Lymphatic Vascular Invasion**				1
No	24/29 (82.76%)	23/30 (76.67%)	47/59 (79.66%)	
Yes	5/29 (17.24%)	7/30 (23.33%)	12/59 (20.34%)	
**Bone Invasion**				1
No	8/29 (27.59%)	8/30 (26.67%)	16/59 (27.12%)	
Yes	21/29 (72.41%)	22/30 (73.33%)	43/59 (72.88%)	

^#^No case showed > 25% large lobules

>25% fibrillary matrix, or pinealocytomatous rosettes

**Fig. 3:**
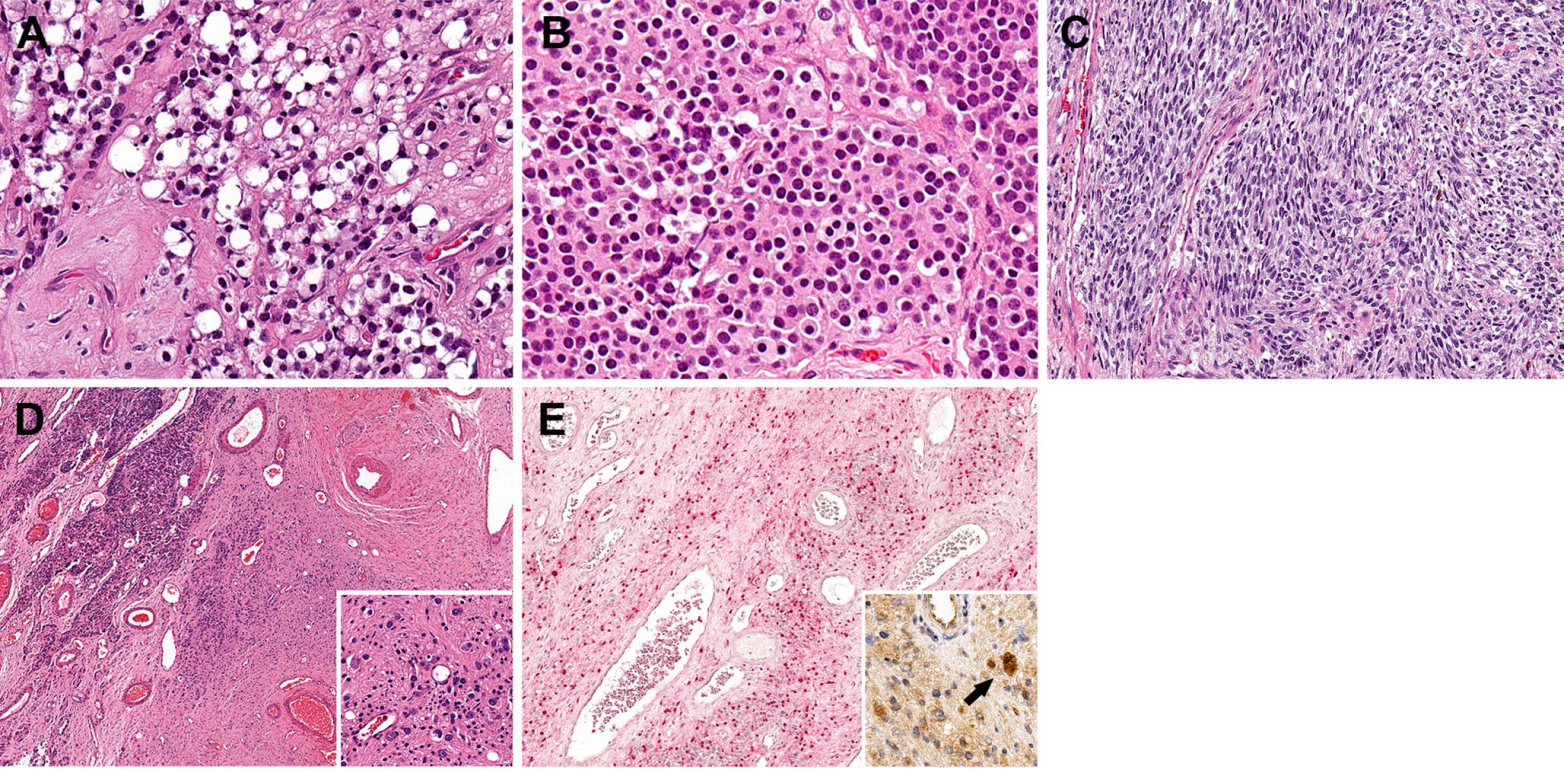
Other morphologies. **A**) Clear cell (40x), and **B**) oncocytic (40x) change were fairly common. **C**) Spindle cell change was somewhat more common in high grade tumors (20x). **D**,**E**: Ganglioneuronal differentiation. **D**) Conventional ONB (top left) transitioning to a spindled stroma (4x). Inset: Scattered embedded ganglion cells (40x). **E**) The spindled stroma is S100 positive. Inset: A subset of ganglion cells was Neuronal nuclear protein positive (40x)

Other key features are illustrated in Fig. 3. Clear cell/oncocytic change (53%, 31/59) and spindling (22%, 13/59) were not uncommon in our cohort, but did not vary significantly between grade groups though spindling was somewhat more frequent in high grade tumors. Similarly, ganglioneuromatous stroma though rare, did not vary between grade groups.

**Fig. 4:**
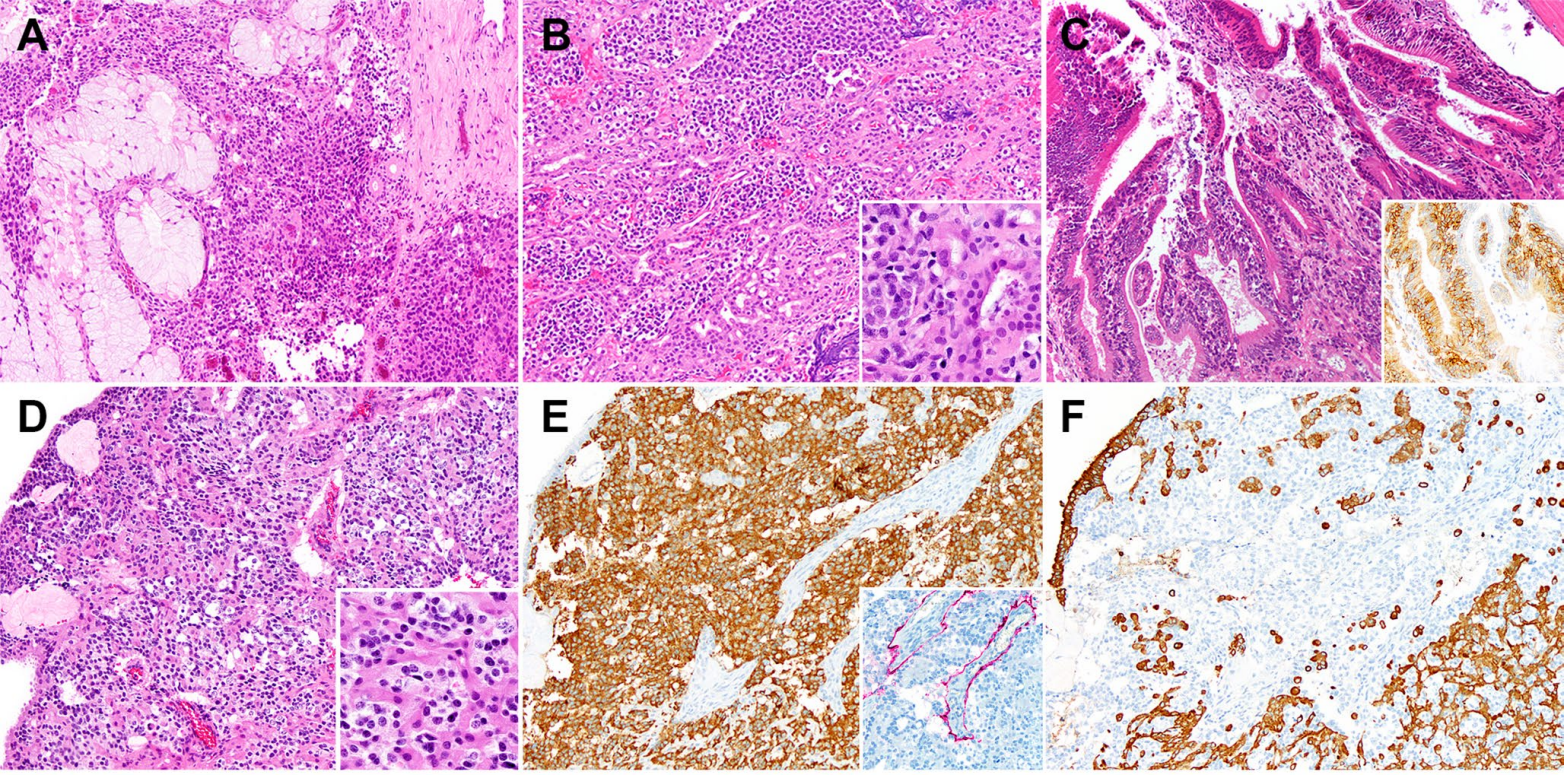
Spectrum of epithelial interactions. **A**) Gland entrapment with glands showing prominent mucous cell predominance (10x). **B**) Gland entrapment with proliferative change with complex back-to-back seromucinous glands (10x). Inset (40x): The nuclei of the seromucinous glands are considerably smaller than those of the tumor (left of inset). **C**) Surface or intraepithelial involvement with accumulation of lesional cells basally (10x). Inset (20x): Intraepithelial component highlighted by an SSTR2 immunostain. **D**-**F**: Olfactory neuroblastoma with divergent epithelial differentiation (olfactory carcinoma). **D**) Anastomosing reticular patterned eosinophilic epithelium admixed with the ONB component (10x, inset: 40x). **E**) The tumor shows synaptophysin staining of the ONB component (10x) along with S100 staining of sustentacular cells (inset: 10x). **F**) Cytokeratin AE1/3 highlights an inverse pattern, restricted to the epithelial component

Only one case (2%) in the low-grade group showed a surface intraepithelial component. Epithelial interactions with seromucinous glands on the other hand were overall somewhat more frequent in the high-grade group, but not significantly so. While divergent epithelial differentiation was exclusive to the high-grade group, only three cases were noted (5%). The spectrum of epithelial interaction is shown in Fig. 4.

Perineural, lymphatic/vascular and bone invasion showed no difference in prevalence in each grade group.

### Outcomes

**Fig. 5:**
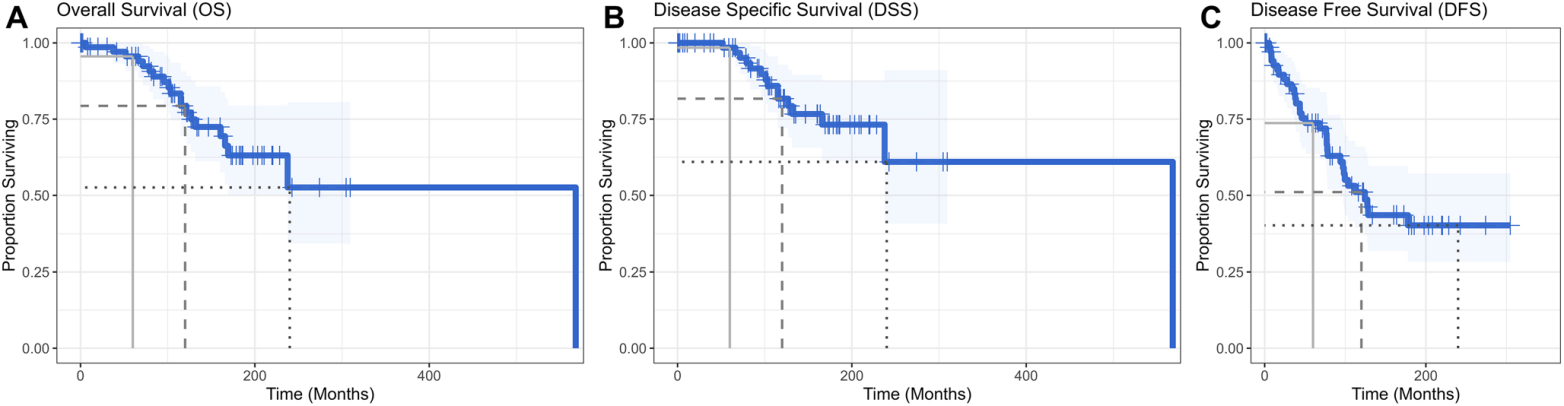
**A**) OS, **B**) DSS, and **C**) DFS in ONB. For each figure, the solid grey drop-down line is 5-year survival, dark grey dashed line is 10-year survival, and black dotted line is 20-year survival

Five-, ten- and twenty-year overall survival (OS), disease specific survival (DSS), and disease-free survival (DFS) are summarized in Table 4 and Kaplan Meier Curves in Fig. 5. Median follow-up on live patients was 121.8 months (range: 0.2-309.4 months). Complete Cox univariable analysis of prognostic variables in terms of OS, DSS, and DFS is shown in Table 5. Significant adverse prognostic features for OS on Cox univariable analysis were: transcranial approach [Hazard Ratio (HR):8.012, (95% confidence interval (CI):1.48-30.487), *p* = 0.02], and brain invasion [HR:4.462 (95%CI:1.067–20.303), *p* = 0.041], with age being nearly significant [HR:1.03 (95% CI:1-1.06), *p* = 0.05]. Significant adverse prognostic features for DSS were: severe nuclear pleomorphism [HR: 8.092 (95% CI:1.329–83.564), *p* = 0.024], combined approach [HR:6.713 (95%CI:1.519–26.229), *p* = 0.015], and transcranial approach [HR:8.092 (95%CI:1.329–83.564), *p* = 0.024], with brain invasion being nearly significant [HR: 5.08 (95%CI:0.97-31.032), *p* = 0.054]. Significant adverse prognostic features for DFS included: severe nuclear pleomorphism [HR:3.81 (95%CI:1.125–12.902), *p* = 0.033], combined approach [HR:4.014 (95%CI:1.177–11.596), *p* = 0.029], transcranial approach [HR:9.687 (95% CI:1.786–36.877), *p* = 0.013], Kadish-Morita Stage C [HR: 8.069 (95% CI:1.11-NA), *p* = 0.035], Kadish-Morita Stage D [HR:17.433 (1.842-NA), *p* = 0.009], intradural invasion [HR:5.553 (95%CI:1.941–21.365), *p* = 0.001], and brain invasion [HR: 7.103 (95%CI:1.64-33.638), *P* = 0.01].

**Table 4:** Survival data

Outcome	5-Year-Survival (95% CI)	10-Year-Survival (95% CI)	20-Year-Survival (95% CI)
OS	96% (91-100%)	79% (69-91%)	53% (34-80%)
DSS	98% (95-100%)	82% (72-93%)	61% (41-91%)
DFS	74% (64-85%)	51% (40-66%)	40% (28-57%)

**Table 5:** Cox univariable analysis of prognostic variables in terms of overall survival (OS), disease specific survival (DSS), and disease-free survival (DFS)

Covariate	OS		DSS		DFS	
	HR(95% CI)	*p*.value	HR(95% CI)	*p*.value	HR(95% CI)	*p*.value
**Age**	1.03 (1-1.06)	**0.05**	1.014 (0.979–1.049)	0.441	1.034 (1.01–1.058)	**0.004**
**Sex**						
Female	Ref	--	Ref	--	Ref	--
Male	1.416 (0.569–3.901)	0.463	0.915 (0.324–2.691)	0.866	1.121 (0.569–2.282)	0.744
**Original Hyams Grade**						
I	Ref	--	Ref	--	Ref	--
II	0.544 (0.167–2.214)	0.362	0.567 (0.144–3.094)	0.467	0.311 (0.102–1.231)	0.089
III	0.499 (0.047–3.169)	0.47	0.676 (0.06–5.263)	0.706	0.393 (0.094–1.783)	0.211
IV	0.49 (0.004–5.08)	0.607	0.706 (0.005–8.762)	0.816	0.214 (0.002–2.241)	0.227
**Modified Hyams Grade** ^**@**^						
II	Ref	--	Ref	--	Ref	--
III	1.925 (0.659–5.776)	0.227	2.485 (0.745–9.072)	0.137	1.952 (0.833–4.725)	0.123
IV	2.063 (0.215–9.821)	0.46	3.009 (0.301–16.303)	0.292	3.12 (0.594–11.066)	0.156
**Architecture** ^**#**^						
> 25% small lobules	Ref	--	Ref	--	Ref	--
> 5% frank infiltration	1.037 (0.113–4.248)	0.967	1.312 (0.141–5.593)	0.764	1.418 (0.376–3.927)	0.564
**Mitoses per 2 mm^2**	1.004 (0.896–1.063)	0.91	1.011 (0.892–1.077)	0.801	1.017 (0.966–1.053)	0.45
**Necrosis**						
None	Ref	--	Ref	--	Ref	--
Punctate	0.651 (0.127–2.174)	0.516	0.868 (0.166–3.061)	0.84	1.699 (0.668–3.95)	0.251
Confluent	1.844 (0.014–14.812)	0.7	2.307 (0.018–19.499)	0.612	0.933 (0.007–7.046)	0.961
**Nuclear Pleomorphism**						
Mild	Ref	--	Ref	--	Ref	--
Moderate	2.092 (0.643–8.499)	0.227	4.408 (0.962–41.845)	*0.057*	1.961 (0.76–5.817)	0.168
Severe	3.44 (0.729–16.232)	0.113	8.092 (1.329–83.564)	**0.024**	3.81 (1.125–12.902)	**0.033**
**Fibrillary Matrix** ^**#**^						
≤ 25% of surface area	Ref	--	Ref	--	Ref	--
None	0.3 (0.002–2.246)	0.308	0.379 (0.003–2.923)	0.434	1.338 (0.354–3.704)	0.628
**Rosette Type** ^**#**^						
Homer Wright	Ref	--	Ref	--	Ref	--
Flexner-Wintersteiner	1.076 (0.008-8.5)	0.96	1.417 (0.011–11.699)	0.821	3.983 (0.419–18.056)	0.187
None	1.594 (0.517–4.448)	0.397	1.675 (0.474–5.27)	0.4	1.841 (0.775–4.203)	0.161
**Calcifications**						
Yes	Ref	--	Ref	--	Ref	--
No	1.396 (0.462–5.486)	0.575	1.055 (0.325–4.28)	0.933	1.988 (0.773–6.385)	0.163
**Procedure**						
EEA	Ref	--	Ref	--	Ref	--
Open	2.011 (0.506–6.214)	0.289	3.932 (0.909–14.816)	0.065	1.178 (0.311–3.295)	0.783
CA	3.971 (0.97-13.008)	*0.054*	6.713 (1.519–26.229)	**0.015**	4.014 (1.177–11.596)	**0.029**
TA	8.012 (1.48-30.487)	**0.02**	12.747 (2.203–57.151)	**0.008**	9.687 (1.786–36.877)	**0.013**
**Kadish-Morita Stage**						
A	Ref	--	Ref	--	Ref	--
B	3.269 (0.265-NA)	0.39	2.041 (0.109-NA)	0.646	1.802 (0.096-NA)	0.706
C	3.74 (0.489-NA)	0.258	3.213 (0.411-NA)	0.334	8.069 (1.11-NA)	**0.035**
D	5.908 (0.48-NA)	0.179	6.108 (0.496-NA)	0.17	17.433 (1.842-NA)	**0.009**
**Nodal disease (within 4 months of treatment)**						
No	Ref	--	Ref	--	Ref	--
Yes	1.726 (0.343–5.539)	0.453	2.51 (0.486–8.538)	0.234	2.542 (0.806–6.306)	0.102
**Number of Sinonasal Compartment Types**						
1	Ref	--	Ref	--	Ref	--
2	0.705 (0.156–4.048)	0.664	1.178 (0.218–11.754)	0.86	1.263 (0.337–6.768)	0.745
3	1.011 (0.258–5.519)	0.988	1.147 (0.212–11.464)	0.883	2.896 (0.862–14.868)	0.089
4	1.629 (0.316–9.801)	0.553	2.728 (0.448–28.187)	0.281	3.462 (0.832–19.307)	0.088
**Maximal Extent**						
Confined to Sinonasal	Ref	--	Ref	--	Ref	--
Early Skullbase	1.625 (0.27–8.362)	0.565	2.261 (0.349–14.654)	0.369	0.927 (0.09–5.637)	0.938
Orbit	0.705 (0.005–7.276)	0.809	0.962 (0.007–11.843)	0.98	2.448 (0.237–14.912)	0.393
Intradural	1.92 (0.562–8.007)	0.305	2.363 (0.593–13.052)	0.231	5.553 (1.941–21.365)	**0.001**
Cutaneous and Soft Tissue	1.134 (0.008–11.91)	0.935	1.541 (0.011–19.378)	0.791	6.818 (0.649–42.906)	0.097
Brain	4.462 (1.067–20.303)	**0.041**	5.08 (0.97-31.032)	*0.054*	7.103 (1.64-33.638)	**0.01**
**Neoadjuvant Radiotherapy**						
No	Ref	--	Ref	--	Ref	--
Yes	1.573 (0.479–4.207)	0.422	2.293 (0.668–6.654)	0.172	2.002 (0.824–4.302)	0.118
**Adjuvant Radiotherapy**						
No	Ref	--	Ref	--	Ref	--
Yes	1.712 (0.601–6.547)	0.336	1.815 (0.542–9.302)	0.361	1.765 (0.76–4.951)	0.198

^@^No case was Modified Hyams Grade I; ^#^No case showed > 25% large lobules, >25% fibrillary matrix, or pinealocytomatous rosettes >25% fibrillary matrix, or pinealocytomatous rosettes

**Fig. 6:**
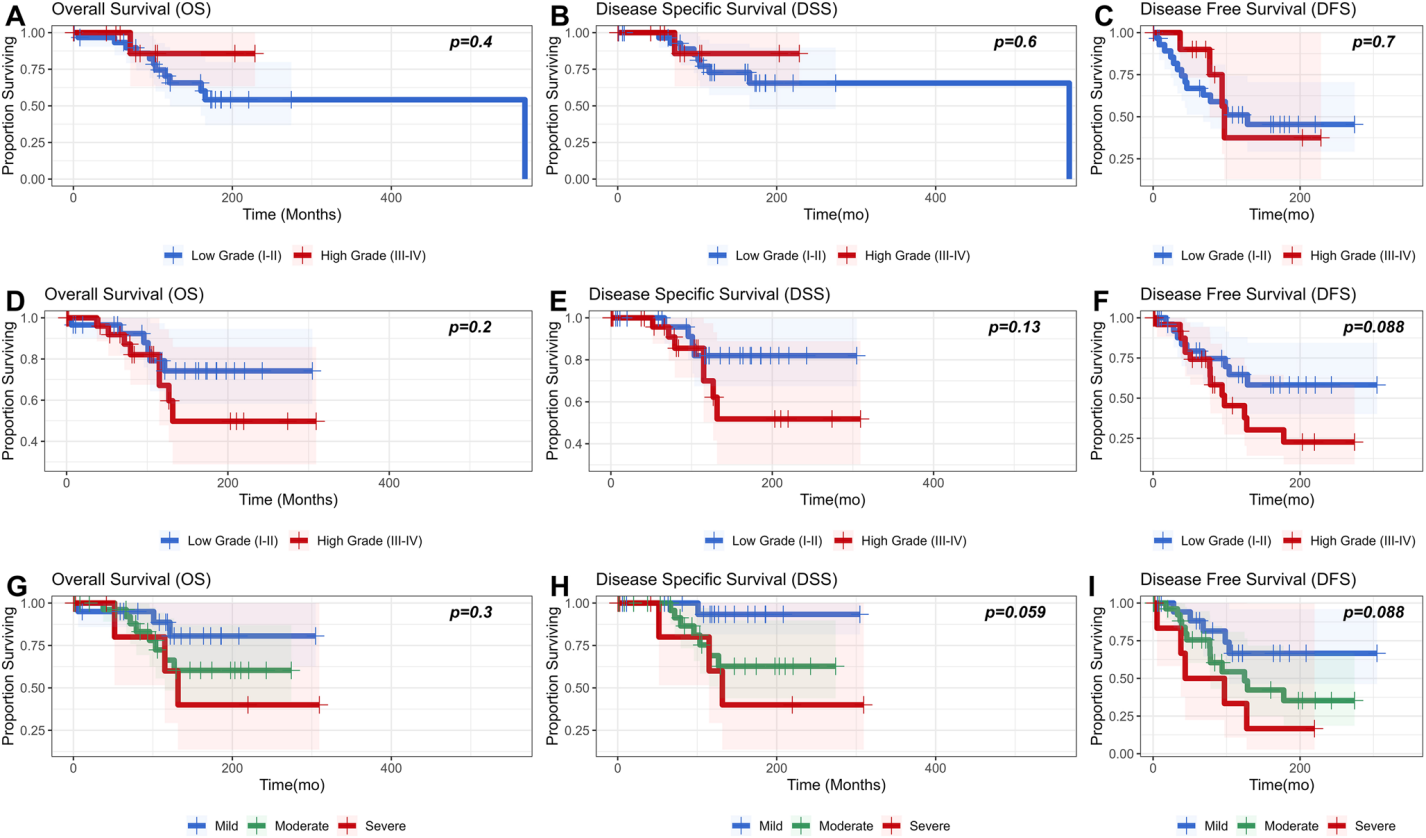
OS, DSS, and DFS by grade and nuclear pleomorphism. **A**-**C**: Original Hyams grade OS, DSS, and DFS respectively. Note the crossover of KM curves for DFS in **C**. **D**-**F**: Modified Hyams grade OS, DSS, and DFS. **G**-**I**: Nuclear pleomorphism OS, DSS, and DFS

Neither Original Hyams Grade nor Modified Hyams grade show significant prognostic value in terms of OS, DSS, or DFS, nor did any individual histologic parameter aside from nuclear pleomorphism noted above. However, Modified Hyams grade did progressively increase in HR for all endpoints in contrast to Original Hyams grade, for which the HRs for grades II-IV were actually lower than the reference (grade I). Dichotomizing into low (grade I-II) and high grade (III-IV) also did not show a significant difference by Kaplan Meier method (Fig. 6) for either grading system, but separation of curves was qualitatively superior for the Modified Hyams grade with DFS between low and high-grade showing a trend towards significance (Log rank *p* = 0.088). By Kaplan Meier method, nuclear atypia across degrees (mild, moderate, severe) showed a trend towards significance for both DSS (*p* = 0.059), and DFS (*p* = 0.088).

The final multivariable Cox regression model for DFS is summarized in Table 6 and includes age, procedure type, nuclear pleomorphism, and Kadish-Morita Stage. Only procedure type (combined and transcranial approaches) remained significant prognosticators [HR:8.616 (95%CI:2.052–37.138), *p* = 0.004, and HR: 17.559 (95%CI:1.727–175.65), *p* = 0.017, respectively]. Kadish-Morita Stage D was nearly significant [HR:14.35 (95%CI:0.963-NA), *p* = 0.054].

**Table 6:** Multivariable Cox regression model for disease free survival

Covariate	HR(95% CI)	*p*.value
**Age**	1.027 (0.993–1.064)	0.118
**Procedure**		
EEA	Ref	--
Open	0.713 (0.058–5.628)	0.758
CA	8.616 (2.052–37.138)	**0.004**
TA	17.559 (1.727–175.65)	**0.017**
**Nuclear Pleomorphism**		
Mild	Ref	--
Moderate	1.549 (0.541–4.88)	0.419
Severe	1.909 (0.265–11.262)	0.506
**Kadish-Morita Stage**		
A	Ref	--
B	1.849 (0.069-NA)	0.722
C	2.698 (0.313-NA)	0.439
D	14.35 (0.963-NA)	*0.054*

## Discussion

Despite the importance attached to Hyams grade for prognosis of ONB, the parameters used to derive grade have not been well scrutinized in the literature [22, 23]. Like other grading systems, the Hyams grading system attempts to frame ONB based on a range of maturation manifested by several key histologic parameters. However, the qualitative nature of Hyams grade leads to subjectivity and inconsistency in prognostic value, with lower grade tumors occasionally showing worse outcomes [11]. This is also compounded by the stringency with which the ONB diagnosis itself is made as well as its interaction with stage as a variable.

While certain morphologic and even immunohistochemical features are implied in prior ONB studies with the acknowledgement that one or more pathologists reviewed the cases, here we explicitly outline criteria integrating morphologic and basic immunohistochemical inclusion criteria and make allowances for divergent epithelial differentiation to accept ‘olfactory carcinoma,’ and our modification of Hyams grade introduces criteria for each parameter.

We found that doing so improves performance of grading beyond the original assigned Hyams grade by providing the expected directionality (i.e. higher grade is actually worse than low grade), and we also reinforce the concept that dichotomizing to low (grade I-II) and high (grade III-IV) grade is more efficient given how sparsely to un-populated the extreme grades are in our cohort [3]. However, this was still insufficient to achieve prognostic significance by any endpoint (OS, DSS, and DFS). We postulate two key factors that contribute to this. First, 59 cases for which a modified Hyams grade could be assigned, while sizeable for an ONB cohort, is still a limited sample size. Second, as this is one of the rare pathologist-driven studies of ONB, the stringency with which we applied the ONB diagnosis is unprecedented likely removing many high-grade tumors that historically would have inflated the prognostic impact of grade as seen in other studies [22].

This finding is not necessarily unexpected given that a relatively recent systematic review does indicate mixed results in terms of prognosis of Hyams grade with 9 studies showing prognostic significance and 9 showing no prognostic significance in terms of OS and even less informative performance for other endpoints (progression free survival and local recurrence) [24]. It must be noted that there was no meta-analysis applied in this systematic review, however it is predicted that it would show considerable heterogeneity and small study bias; only a few included studies exceeded 100 patients [3, 11, 22]. A subsequent multicenter study of 256 patients that included at least a subset of the current cases did show univariable prognostic significance for grade in terms of DFS (despite the absence of pathologist input/review) and improvement of model performance when integrating grade into stage groupings as assessed by C-index [7].

Despite the improvements to Original Hyams grade, the impact is not profound. We thus attempted to determine the parameters that contributed most to grade in terms of both application and prognosis. As expected, pleomorphism, mitotic rate and absence of rosettes were discriminatory between low- and high-grade tumors, though other expected features such as necrosis and growth pattern were not, after p-value adjustment. However, only pleomorphism on its own was a univariable prognosticator. Thus, it appears that while useful descriptively, most Hyams grade parameters are superfluous. The lack of prognostic value for mitotic rate and necrosis is surprising but potentially explainable by technical considerations. As our cohort is endoscopic surgery predominant spanning a large time-period with different gross and histologic specimen processing protocols throughout the decades, the resulting variable specimen quality, specifically crush artifact and excessive fragmentation, make it difficult to accurately capture these parameters. While atypia is also similarly prone to artifact (usually overinterpretation), less preservation may be required to assess this.

Most studies are limited in the scrutiny of Hyams grading components. In the study by Van Gompel et al. [22], a mitotic cutoff of 5 mitoses per 10 high power fields was used to characterize low grade (I/II) vs. high grade (III/IV) tumors and severe anaplasia and necrosis were considered characteristic of grade IV in particular. Gallagher et al. [23] were arguably the first to methodically evaluate individual components of Hyams grade and additional parameters such as spindling, and epithelial interaction (‘gland hyperplasia’). In fact, their novel grading system with spindling, gland hyperplasia and necrosis performed better than Hyams grade. In our hands, spindling did correlate somewhat with higher grade but was not significantly so after p-value correction. Gland hyperplasia was tangentially related to our rubric of epithelial interaction and likely fit our category of proliferative gland entrapment, which can be likened to the tumor-associated ductular proliferation that intermingles with the endocrine component in ductulo-insular tumors of the pancreas [25]. While Gallagher et al. [23] note this as a favorable characteristic, we saw no difference by grade, and in fact, this was slightly more frequent in higher grade tumors.

We introduced an assessment of a spectrum of epithelial interactions to determine if there was any relationship to divergent epithelial differentiation. The current popular designation for tumors with divergent epithelial (usually glandular) differentiation is ‘olfactory carcinoma’ [26] though even this can be subdivided into tumors with distinct ONB and carcinomatous components (akin to the mixed neuroendocrine-non-neuroendocrine neoplasm (MiNEN) nomenclature recently introduced in the 5th edition WHO Classification of Endocrine and Neuroendocrine Tumors [27]) and those that have the appearance of ONB [28], almost invariably high grade and show only subtle epithelial characteristics, often only highlighted by immunohistochemical stains. Recurrent molecular alterations, most commonly in genes affecting the Wnt pathway, have been identified in a subset of these tumors, suggesting that molecular profiling may offer additional insight into their classification [29]. We found that while various epithelial interactions were common, divergent epithelial differentiation/olfactory carcinoma is uncommon at ~ 5% in our in-house cohort (we do have several more in our consult files but not within the scope of this study) and as noted in a large multi-institutional series [26], restricted to high grade tumors. However, we were unable to make a case for a transitional relationship between this and other epithelial interactions. Given the rarity of divergent epithelial differentiation, formal outcomes analysis was not performed. However, the patients were all female (ages 15, 31 and 51). Only one patient died of disease at 127 months, while the other two patients are alive at 54 and 66 months.

Similarly, *de novo* ganglioneuromatous stroma is uncommon (slightly under 7%) and evenly distributed between low and high grade. Ganglioneuromatous differentiation had been previously described in the setting of prior treatment [30] but none of our cases had neoadjuvant treatment. Isolated ganglion-like cells have also been noted in 11% of olfactory carcinomas [26].

While our focus was specifically on grading and histologic parameters, we did integrate these with clinical variables, and several interesting findings emerged. Given the extent of follow-up in this cohort we were able to confirm that patients remain at risk for events well beyond 10 years. We confirm that procedure type and extent of disease, whether via Kadish-Morita Stage or specific structures involved (i.e. intradural, brain), are the main predictors of outcome. In this cohort, endoscopically resected cases had a better prognosis even on multivariable analysis. This is in line with a recent NCDB analysis [31]. The rationale for better outcomes likely goes beyond just selecting for less aggressive cases as the NCDB study demonstrated shorter hospital stays and thus less morbidity and improved overall level of care. A multi-institutional study including cases from our institution of stage matched open vs. endoscopically treated ONB also showed improved outcomes in the latter [32], showing that margin control with an endoscopic approach was superior.

Several staging systems now exist. The Dulguerov T categorization is likely superior to the Kadish-Morita staging system [7], but the latter is what was readily available to us. We do document that Kadish-Morita stage performance is largely driven by Stage D, namely metastasis. Age was prognostic in terms of DFS (and as expected OS) but not significantly so on multivariable analysis. While incorporation of more detailed structural involvement would have been desirable for prognosis, the sample size and number of events limits the number of variables to include without overfitting the model. Dural invasion has been identified as an adverse prognosticator in terms of survival and regional recurrence [32, 33].

Thus, in summary, while refinement of Hyams grading in ONB improves performance, the impact is not exceptionally profound. We also highlight the potential to streamline grading into a limited set of parameters, though in this cohort, only nuclear pleomorphism was a significant univariable prognosticator. We also reinforce the importance of procedure type and disease extent in terms of prognosis on this cohort with extended outcome data.

## Data Availability

No datasets were generated or analysed during the current study.
